# Impact of HIV pre-exposure prophylaxis (PREP) on health-related quality of life (QOL) of patients followed up at a reference center in São Paulo, Brazil

**DOI:** 10.1016/j.clinsp.2024.100419

**Published:** 2024-07-08

**Authors:** Michelle Kaoru Takada-de-Souza, Camila de Melo Picone, Vivian Iida Avelino-Silva, Angela Carvalho Freitas, Aluisio Cotrim Segurado

**Affiliations:** Departamento de Medicina Tropical e Infectologia, Faculdade de Medicina, Universidade de São Paulo, São Paulo, SP, Brazil

**Keywords:** HIV, Pre-exposure prophylaxis, PrEP, Quality of life

## Abstract

**Background:**

HIV Pre-Exposure Pophylaxis (PrEP) is provided free of charge by the Brazilian national health system. Though effective in preventing HIV infection, little is known about its impact on the health-related Quality of Life (QoL) of users.

**Objective:**

The present study aimed at assessing the impact of PrEP on the QoL of its users.

**Methods:**

Prospective cohort study with 114 HIV-negative participants aged 18 years or older. Participants' QoL was assessed before starting PrEP and after 7 months of use, using the self-responsive WHOQOL-bref questionnaire. Sociodemographic and behavioral aspects were described and the Wilcoxon signed-rank test with p ≤ 0.05 was considered statistically significant.

**Results:**

Improvement was seen in QoL scores for the environment domain (p = 0.02), which addresses feeling of physical safety, access to information and health services, and participation in leisure activities. Furthermore, participants reported improved satisfaction with their sex life, when questioned about the social relationships domain. There was no statistically significant change in the global QoL score, in the global health score, in the physical and psychological domains, nor in the total score for the social relationships domain. As for their socio-demographic profile, most participants were white and highly educated young cisgender men who have sex with men. 76.3% had unprotected sex in the 3 months before starting PrEP. 60.5% had reported substance use: marijuana (42.1%), club drugs (35.1%), and poppers (20.2%).

**Conclusions:**

This study unveiled that PrEP benefited our cohort beyond its effectiveness in preventing HIV infection, having improved environmental aspects of QoL and self-satisfaction with sex life.

## Highlights

• PrEP has benefits beyond HIV prevention.
• PrEP improves users’ quality of life.
• PrEP improves self- satisfaction with sex life.

## Introduction

According to international recommendations, HIV prevention is most effectively accomplished with a combined approach that comprises the use of prophylactic technologies associated with structural interventions to mitigate vulnerability to infection.^1^ In this scenario, recognition of intersubjective contexts is needed to adapt interventions to the individual's socioeconomic status, values and expectations, cultural backgrounds, and interpersonal relationships. As such, the development of tailored self-care plans may enable the adoption of safer sexual behaviors best suited to each person's life.^2^,^3^

In accordance with this action plan, PrEP was approved in Brazil as a preventive tool in May 2017, to be provided free-of-charge within the national unified public health system (SUS), following a standardized protocol.^4^,^5^ The strategy consists of administering a combination of antiretroviral drugs (tenofovir and emtricitabine) prior to viral exposure to reduce HIV risk. It targets key populations, characterized as under high vulnerability to viral acquisition, considering the local epidemiological pattern of HIV transmission, namely, Men who have Sex with Men (MSM), Transgender people (Trans), sex workers, and serodiscordant couples.

Despite clear evidence of its efficacy and effectiveness in preventing HIV infection,^6^,^7^ little is known so far about additional potential benefits of PrEP, including its effect on the Quality of Life (QoL) of populations using it. Such knowledge would help understand to what extent this policy, incorporated in SUS, contributes to the comprehensive care of vulnerable populations, providing a patient-centered means of sexual health promotion that may affect their QoL.

It is widely recognized that the practical success of a given health intervention depends not only on its technical effectiveness but also on the subjective perceived impact it yields in the life of the recipient, motivating him/her to adhere to it and effectively incorporate it in one's routine. The existence of additional benefits, with an improvement in lived experiences as a whole, serves as an important motivator, given that, often, scientifically proven technical effectiveness alone is not capable of encouraging people to adopt the recommended medical care.^8^

Given these considerations and the fact that a positive impact on QoL may change the way patients relate to medical interventions,^8^ the authors conducted this study to assess the impact of PrEP on the QoL of patients using it.

## Material and methods

This prospective observational cohort was carried out at the HIV outpatient clinic (SEAP), affiliated with the Division of Infectious and Parasitic Diseases, Hospital das Clínicas, at the School of Medicine of the University of São Paulo in São Paulo, Brazil. Recruitment of participants occurred between July 25, 2018 and May 3, 2019, based on a convenience sampling strategy that included different PrEP delivery times (morning, afternoon and evenings) in operation at the clinic.

Patients who complied with national guidelines for PrEP distribution within the national health system were considered eligible for inclusion in the study, namely, individuals aged 18 years or older, under high vulnerability of HIV acquisition. The authors excluded those who reported previous use of PrEP.

The authors extracted participants’ sociodemographic data and information about HIV exposure at baseline from the national electronic database that registers all individuals using PrEP within SUS (SICLOM-PrEP). On the day of inclusion, a member of the research team applied a complementary standardized questionnaire to all study participants to obtain information about age, gender identity, familiar income, number of people supported by the income, and whether the participant was in a serodifferent sexual partnership or not.

To assess patients’ QoL the authors used a short quantitative tool developed by the World Health Organization (WHOQOL-bref) in its previously validated Portuguese translation.^9^ Composed of 26 questions, it addresses individual perceptions about QoL, health, and other aspects experienced in the two weeks prior to questionnaire completion, yielding a comprehensive QoL profile. The authors applied the questionnaire at baseline and after 7 months on PrEP. To provide a broad assessment, 24 of the 26 questions were used to gather data related to facets incorporated in the four QoL domains, namely:

1) Physical domain: activities of daily living; dependence on medicinal substances and medical aid; energy and fatigue, mobility, pain and discomfort; sleep and rest; work capacity.
2) Psychological domain: body image and appearance; negative feelings; positive feelings; self-esteem; spirituality, religion, personal beliefs; thinking, learning, memory and concentration.
3) Social Relationships domain: personal relationships; social support; sexual activity.
4) Environment domain: financial resources; freedom, physical safety and security; health and social care: accessibility and quality; home environment; opportunities for acquiring new information and skills; participation in, and opportunities for recreation/leisure activities; physical environment (pollution/noise/traffic/climate); transport.

The questionnaire is organized under a Likert-type response scale, with results in scores ranging between 1 and 5 for each question. It was thus possible to calculate, according to the standardized procedures proposed by the WHOQOL Group, four different domain scores, yielding a final 0‒100 scale. Responses to the remaining two questions, which are the first two of the questionnaire and not related to any particular QoL domain, were considered separately to address (1) the individual's overall perception of QoL and (2) one's health, also generating scores from 0 to 100. Higher scores denote higher QoL.^10^, ^11^, ^12^

Patients’ adherence to PrEP medication was also evaluated using drug dispensation data obtained from the clinic pharmacy records and using a self-response questionnaire, applied to participants at 4 and 7 months of follow-up under PrEP. For analysis the authors defined “adequate adherence to PrEP” as having a self-report of taking at least 8 pills of PrEP medication in the two weeks prior to the 4- and 7-month follow-up consultations for males, and of having taken all 14 pills for females. In addition, self-reported adherence had to be consistent with the pharmacy's dispensation records for adherence to PrEP to be considered adequate.

Data obtained from SICLOM-PrEP, the study complementary questionnaire, the self-reported adherence questionnaire, the clinic pharmacy dispensation records, and from WHOQOL-bref were all transcribed to a study database, using the REDCap (Research Electronic Data Capture) system.^13^

To characterize the study population, categorical variables of interest are presented using absolute numbers and frequencies and quantitative variables with central trend and dispersion measures. QoL assessment results are expressed in scores, resulting in four domain scores and one individual score for each of the two questions, which assess the individual's overall perception of QoL or of one's health.

To test the hypothesis that there was no difference between QoL scores, assessed at baseline and after 7 months on PrEP, the authors used the Wilcoxon Sign-rank test. For this analysis, the authors adopted the “Intention To Treat” (ITT) strategy.^14^ Statistical analysis was conducted using a bidirectional α of 0.05, with computational support of the Excel 2016® (Microsoft Office) and Stata (version 15.1) softwares.

The study was approved by the Institutional Review Board as protocol #90859418.8.0000.0068. All participants provided autonomous, free and informed consent prior to inclusion in the study. Moreover, the authors ensured subject anonymity and data confidentiality throughout the study.

## Results

**Fig. 1:**
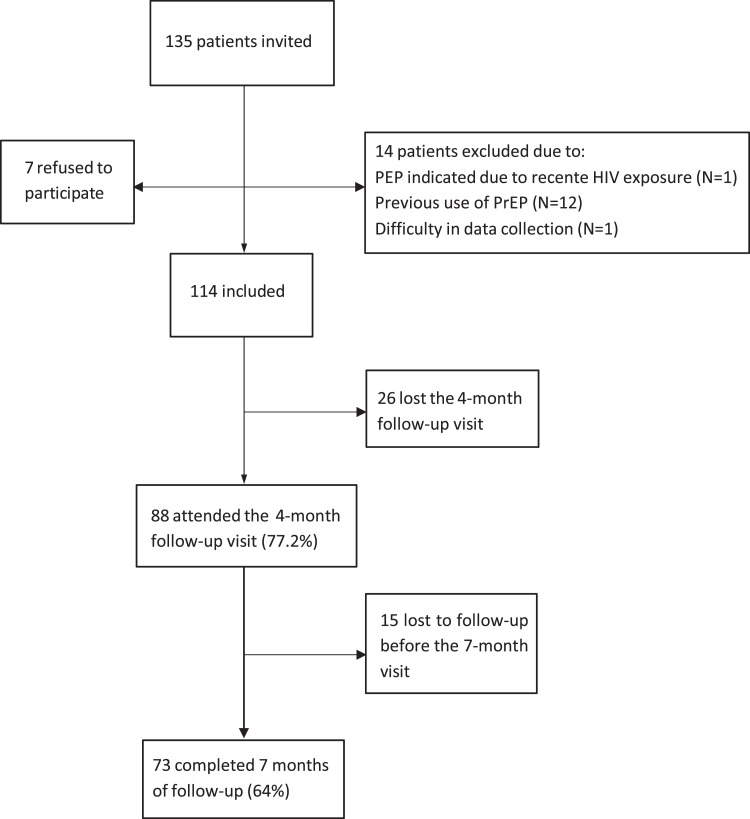
Participant inclusion in the study cohort 2018 – 2020.

Of the 135 eligible patients the authors invited to participate in the study, 7 refused. Alleged main reasons for refusal included lack of time (n = 4) or interest (n = 2) and refusal to provide electronic data (n = 1). Additionally, 14 patients were excluded because of previous use of PrEP (n = 12), high difficulty in answering the questionnaire (n = 1), or because of having been started on HIV Post-Exposure Prophylaxis (PEP) after recent viral exposure (n = 1). 41 (36%) patients were lost from follow-up. Our cohort thus comprises 114 participants, 73 (64%) of whom fully completed the proposed 7-month follow-up, as shown in Fig. 1.

The sociodemographic characteristics of our cohort are summarized in Table 1. 106 (93%) participants were Men who have Sex with Men (MSM) and 21 (18.8%) reported having a serodiscordant sexual partner regardless of sexual orientation. Most participants self-reported being white (71.9%), identified themselves as cisgender men (97.4%), and had high schooling (95.6% reported 12 or more years of education). The cohort had a median age of 30 years (interquartile range, IQR 27‒34), and presented a high-income pattern.

**Table 1:** Sociodemographic characteristics of the study cohort at baseline (n = 114), 2018‒2020.

Variables	n	%
Median age (IQR)	30 (27‒34)	‒
Sex assigned at birth		
Male	111	97.4
Female	3	2.6
Skin color		
White	82	71.9
Brown	26	22.8
Black	6	5.3
Median per capita income in Brazilian reais (IQR)^a^	4000 (2000‒7000)	‒
Schooling (years of education)		
≥12	109	95.6
8‒11	4	3.5
4‒7	1	0.9
Country of birth		
Brazil	112	98.2
Other	2	1.8
Site of birth^b^		
São Paulo	73	64.6
Other Brazilian states	38	33.6
Foreign countries	2	1.8
Gender identity		
Cisgender man	111	97.4
Cisgender woman	3	2.6
Sexual orientation		
Homosexual	97	85.1
Bisexual	9	7.9
Heterosexual	8	7.0

IQR, Interquartile Range.

a Data missing for 3 individuals.

b Data missing for 1 individual.

Behavioral and vulnerability characteristics of the study cohort at baseline are depicted in Table 2. Regarding HIV prevention, 16 (14%) patients of the cohort reported using condoms in less than 50% of sexual contacts in the 3 months before starting PrEP. Substance use was reported by 69 (60.5%) participants, more often consuming marijuana (42.1%), club drugs (35.1%) and poppers (20.2%). Only 4 (3.5%) informed having used injected drugs and 1 (0.9%) had shared needles/syringes for injection of anabolic steroids, hormones, or silicone in the previous 3 months.

**Table 2:** Behavioral and vulnerability characteristics of the study cohort at baseline (n = 114). 2018‒2020.

Variables	n	%
PrEP indication		
Men who have sex with men (MSM)	106	93
HIV serodifferent sexual partnership^a^	21	18.8
Sex worker	4	3.5
Trans people	‒	‒
Motivation for seeking PrEP^b^		
Sensitized by printed communication/internet/friend/own decision	89	78.8
Recommended by a health care professional	24	21.2
Recommended by an NGO activist	‒	‒
Reproductive intentions		
No	108	94.7
Yes	6	5.3
Alcohol use in the previous 3-months (≥5 shots in 2 hours)	74	64.9
Substance use (previous 3-months)		
Marijuana	48	42.1
Club drugs (ketamine, ecstasy, LSD, GHB, bath salts, etc.)	40	35.1
Poppers (amyl or alkyl nitrites)	23	20.2
Erection stimulants	22	19.3
Cocaine	21	18.4
Solvents	3	2.6
Crack	2	1.8
None of the above	45	39.5
Injected drug use (without medical prescription)		
Never	110	96.5
Yes, but not in the previous 3-months	3	2.6
Yes, in the previous 3-months	1	0.9
Used shared instruments to inject anabolic steroids/hormones/silicone (previous 3-months)	1	0.9
Condom use (previous 3-months)		
Never	5	4.4
< 50% of intercourses	11	9.6
50% of intercourses	15	13.2
> 50% of intercourses	56	49.1
Always	27	23.7

NGO, Non-Governmental Organization.

a Data missing for 2 individuals.

b Data missing for 1 individual.

Among the 73 patients who completed the 7-month follow-up, 55 (75.3%) had their adherence to PrEP assessed as adequate.

WHOQOL-bref scores at baseline and after a 7-month follow-up under PrEP are shown and compared in Table 3, according to the four QoL domains and the two questions not related to any particular QoL domain.

**Table 3:** Quality of Life (QOL) scores, assessed with the WHOQOL-bref tool, at baseline (n = 114) and after 7 months of follow-up under PrEP (n = 73), 2018‒2020.

Domain/Question	QoL at baseline, Median (IQR)	QoL after 7-months under PrEP, Median (IQR)	p-value^a^
Domain 1 (physical)	75.0 (64.3‒85.7)	75.0 (67.9‒85.7)	0.55
Domain 2 (psychological)	70.8 (58.3‒79.2)	70.8 (62.5‒79.2)	0.48
Domain 3 (social relationships)	66.7 (58.3‒75)	75.0 (58.3‒83.3)	0.14
Domain 4 (environment)	68.8 (59.4‒78.1)	71.9 (62.5‒81.3)	0.02
Question 1 (Overall QoL perception)	75.0 (75.0‒100)	75.0 (75.0‒75.0)	0.51
Question 2 (Overall health perception)	75.0 (75.0‒75.0)	75.0 (75.0‒75.0)	0.44

IQR, Interquartile Range.

a Wilcoxon Sign-rank test.

A significant increase in QoL was found for domain 4 (environment) (p = 0.02). The increase in the QoL score for domain 4 after starting PrEP was not associated with age, per capita income, skin color, or report of substance use. In contrast, no difference in QoL scores was shown for domains 1 (p = 0.55), 2 (p = 0.48), 3 (p = 0.14), and for questions 1 (overall perception of QoL; p = 0.51) and 2 (overall perception of one's health; p = 0.44) in our cohort.

As previously described, PrEP was not shown significantly associated with a change in QoL scores for domain 3 in our cohort. This particular QoL domain addresses social relationships, including aspects related to personal relationships, social support, and sexual activity. Nevertheless, to better characterize participants’ satisfaction with sex life, the authors further investigated responses among the 73 patients who completed the study follow-up to question 21 of the WHOQOL-bref, which specifically addresses one's satisfaction with sex life: “How satisfied are you with your sexual life?”.

Using a Likert scale, ranging from 1 (very dissatisfied) to 5 (very satisfied) with one's sex life, the authors found that among the 27 participants who were not satisfied with their sex lives at baseline, i.e., those who chose alternatives 1 (very dissatisfied), 2 (dissatisfied) or 3 (neither dissatisfied nor satisfied), 12 (44%, 95% CI 25%‒65%) informed being satisfied with their sexual lives after 7 months on PrEP. In contrast, out of the 46 who were satisfied with their sex lives at baseline, i.e., those who chose response alternatives 4 (satisfied) or 5 (very satisfied), only 10 (22%, 95% CI 11%‒36%) reported dissatisfaction at the 7-month follow-up visit.

No statistical difference in sociodemographic and behavioral characteristics was found between PrEP users in our cohort who completed or were lost from follow-up. The authors evaluated age, skin color, genital organ of birth, schooling, per capita income, sexual orientation, condom use, and substance use (marijuana, cocaine, club drugs, poppers, crack and solvent). In addition, scores for questions 1 and 2 and for all four QoL domains at baseline did not differ significantly between the two groups.

Likewise, there was no statistically significant difference between patients with adequate or inadequate adherence to PrEP, regarding sociodemographic and behavioral characteristics or WHOQOL scores for questions 1 and 2, and for QoL domains 1 to 4.

## Discussion

The present study, carried out at a university-affiliated reference HIV clinic located in the largest Brazilian city has demonstrated that PrEP improved the QoL of its users. Results revealed a statistically significant increase in the QoL scores for domain 4 (p = 0.02). To better interpret this, it is first necessary to understand what WHOQOL-bref domain 4 is about. Composed of eight different questions, it addresses different aspects related to the environment the respondent is exposed to and their consequences. This includes the availability of financial resources, a feeling of physical security, access to health care, housing conditions, opportunities to acquire new information and skills, participation and opportunity for recreation and leisure activities, and satisfaction with transportation.^11^

The authors thus interpret that PrEP can contribute to reducing one's vulnerabilities, as it attracts the user to the health system and gives him/her the opportunity to access close medical monitoring, screening for sexually transmitted infections and their treatment, if necessary, vaccination, and also to receive important educational information for self-care. This is particularly relevant in the Brazilian context, where PrEP as an HIV prevention tool is offered free of charge through the public health system, following a standardized protocol.^5^ As a result, PreP users feel empowered and may experience an improvement in the feeling of physical security, which translates into greater freedom to engage in recreational, leisure and sexual activities, in addition to a perception of better access to health services, as well as to a valuable opportunity to acquire information for health promotion.

Our data interpretations coincide with the findings of the qualitative study by Bistoquet et al.,^15^ focused on the analysis of the main motivations to seek PrEP. In that study, interviewees reported that in addition to the fear associated with HIV infection, the benefit of regular, personalized medical follow-up and the desire to take care of one's own health were the main stimuli for seeking PrEP. They also reported improved sex life, and increased sense of freedom, in addition to satisfaction with regular screening for HIV and other STIs.

In this context, the provision of welcoming and comprehensive care at the HIV clinic may have contributed to the improved scores for QoL domain 4 observed in our cohort. In fact, when these environmental characteristics are not present, PreP discontinuation rates tend to be higher, with inadequate management of associated risk, as discussed by Carvalho et al.^16^

The WHOQOL-bref questionnaire has been previously used by Liu et al.^17^ in a cross-sectional study to assess QoL of 374 young MSM with negative or unknown HIV serostatus living in two U.S. metropolitan areas and to determine whether specific QoL domains were associated with participants’ demographic, psychosocial, and behavioral data, and with their engagement in HIV prevention. Among other results, authors concluded that higher physical/psychological and environmental QoL scores were associated with greater likelihood of HIV testing and PrEP use. Differences in methodological approach, however, preclude full comparison with our results. Kapadia et al.^18^ evaluated 591 participants of a randomized phase 2 PrEP safety trial and found that participants’ EQ-5D-3L mean scores at baseline were similar to those found in the U.S. general population of comparable age and remained stable over time. Differences in the sociodemographic profile of participants, as well as in the QoL assessment tool used may account for the contrasting results found in our study.

Another important finding in our study was improvement in self-satisfaction with sex life after being started on PrEP use. Similar evidence was provided by Van Dijk et al.,^19^ who showed improvement in quality of sex life in the first months under PrEP, accompanied by reduction of fear of acquiring HIV and increased interest in experiencing new sexual practices among PrEP users in the Netherlands. Likewise, Montgomery et al.^20^ described improvement in sexual satisfaction among MSM using PrEP in two urban clinics in the United States. In the study by Bertevello,^21^ which evaluated the effects of PrEP on the quality of sexual life and mental health of Brazilian users, an improvement in sexual parameters was verified, including relevant effects related to libido, arousal, erection and sexual satisfaction. In that cohort, a reduction in the interference of fear of HIV during and after sexual intercourse and improved access to health care were also observed. Lastly, in the IPERGAY trial, Mabire et al.^22^ assessed how pleasure-seeking behaviors among MSM play a role in HIV prevention and in the quality of their sexual life, and how this can result in PrEP initiation. Based on data collected in semi-structured interviews, the authors concluded that PrEP reduced patients’ anxiety and fear of HIV acquisition, promoted better enjoyment of intimacy, and ultimately led to improvement in the quality of their sex lives.

In contrast to the reported increase in QoL scores for domain 4 (environment), no statistically significant changes were seen in scores for the other QoL domains in our cohort. The authors believe scores for domains 1 (physical aspects), 2 (psychological aspects), and 3 (social relationships) probably remained unaltered because participants were physically healthy at all times in our study and may not have experienced significant disfavorable feelings regarding how they enjoyed life, accepted their physical appearance and engaged in interpersonal relationships at baseline.

Regarding the limitations of our study, it is important to highlight that since it is a single-center study, generalization of its results is challenging. However, the authors must recognize that it raised a relevant research question that can be further addressed in future multicenter studies. Additionally, 36% of participants enrolled in our cohort were lost to follow-up before the 7-month QoL assessment and could not undergo the 7-month QoL assessment. Nevertheless, having used the intention-to-treat approach in data analysis, and showing that PrEP users who were lost to follow-up did not differ significantly from those who were retained throughout the study in terms of sociodemographic and behavioral profiles, or in adherence to PrEP, make us believe that selection bias is improbable.

## Conclusion

The authors conclude that this study contributes to recognizing that PrEP benefits go beyond its biological effectiveness in preventing HIV acquisition. Our results unveil among PrEP users an improved perception of environmental aspects of QoL and of self-satisfaction with sex life. As such, our findings can help health professionals change the way PrEP is presented to potential users, adding new considerations to clinical decision-making and in the way results of this intervention are evaluated.

## Statements and declarations

Ethics approval: The present study was approved by the Institutional Review Board/Ethics Committee – protocol #90859418.8.0000.0068. It was carried out in accordance with the ethical standards laid down in the Declaration of Helsinki. Informed consent was obtained from all participants included in the study.

Consent to participate: All subjects provided written informed consent.

Consent to publish: Not applicable.

Data availability statement: The datasets generated during and/or analyzed during the current study are available from the corresponding author upon reasonable request.

## Authors’ contributions

Michelle K. Takada-de-Souza contributed to the study conception and design, data collection, data analysis, data interpretation, manuscript writing and revision. Aluisio Cotrim Segurado contributed to the study conception and design, data analysis, data interpretation, manuscript writing and revision. Camila de Melo Picone contributed to the study conception, data collection, data interpretation and manuscript revision. Vivian Iida Avelino-Silva contributed to study conception, data analysis, data interpretation, manuscript writing and revision and Angela Carvalho Freitas contributed to data interpretation, manuscript writing and revision. All authors read and approved the final manuscript.

## Declaration of competing interest

Authors Takada-de-Souza MK, Picone CM, Avelino-Silva VI, Freitas AC and Segurado AC have no relevant financial or non-financial interests to disclose.
